# U.S. young adults’ awareness of the Master Settlement Agreement and cigarette industry practices and their associations with electronic cigarette industry and health risk perceptions

**DOI:** 10.1186/s12889-023-15520-2

**Published:** 2023-03-31

**Authors:** Lilianna Phan, Kelvin Choi

**Affiliations:** Division of Intramural Research, National Institute on Minority Health and Health Disparities, 9000 Rockville Pike, Bethesda, MD 20892 USA

**Keywords:** Electronic cigarettes, e-cigarettes, Industry denormalization, Communication, Prevention, Young adults

## Abstract

**Background:**

The lawsuit that led to the U.S. Master Settlement Agreement (MSA) exposed the cigarette industry’s deceptive marketing practices, which changed population perceptions about the cigarette industry and helped prevent cigarette smoking. The cigarette industry now owns many electronic cigarette (e-cigarette) companies and make their own e-cigarettes. Given that the MSA occurred in previous decades, many millennial and generation Z young adults may not know about the MSA and the cigarette industry’s marketing practices. It is unknown whether awareness about the MSA and cigarette industry practices may influence these young adults’ e-cigarette industry and e-cigarette health risk perceptions, which may inform e-cigarette prevention efforts.

**Methods:**

Cross-sectional data were collected from a U.S. sample of tobacco-naïve young adults, 18–30 years-old, susceptible to e-cigarette use (*n* = 1,329) through an online panel service in August 2021-January 2022. Participants reported their demographic characteristics, awareness of the MSA, awareness of cigarette industry practices, e-cigarette industry perceptions, and e-cigarette health risk perceptions. We examined the relationships between awareness of the MSA and cigarette industry practices with e-cigarette industry and e-cigarette health risk perceptions using multivariable linear regressions, adjusted for demographic characteristics.

**Results:**

Overall, 36.2%, 24.1%, and 39.3% of participants had heard of the MSA and knew a lot about it, had heard of the MSA, but did not know much about it, and did not hear of the MSA, respectively. On average, participants were aware of 5.2 (SD = 3.0) of the 11 cigarette industry practices included. Hearing about the MSA and knowing a lot about it and awareness of more cigarette industry practices were associated with less positive e-cigarette industry and higher e-cigarette health risk perceptions, whereas having heard of the MSA but not knowing much about it was associated with more positive e-cigarette industry and lower e-cigarette health risk perceptions.

**Conclusions:**

Findings suggest that increasing comprehensive awareness of the MSA and cigarette industry practices may influence young adults’ e-cigarette-related perceptions, and may importantly prevent detrimental information gaps about the cigarette industry. Future research should investigate the potential impact of increasing awareness of the MSA and cigarette industry practices in changing e-cigarette-related perceptions, which may help prevent e-cigarette use.

**Supplementary Information:**

The online version contains supplementary material available at 10.1186/s12889-023-15520-2.

## Introduction

Young adults have the highest rates of electronic cigarette (e-cigarette) use worldwide [1, 2]. E-cigarettes contain harmful chemicals including nicotine; therefore, e-cigarette use during young adulthood poses substantial health harm and addictive risks [3]. For example, e-cigarette use among young adults who never smoked cigarettes is associated with long-term consequences to the developing brain, poorer respiratory health [4, 5], and subsequent uptake of other tobacco products including cigarettes [3, 6, 7], making e-cigarette use prevention a significant public health agenda [3].

The 1998 U.S. Master Settlement Agreement (MSA), resulting from a historic civil ligation settlement between the Attorneys General for 46 U.S. states, five U.S. territories, and the District of Columbia and the four largest cigarette manufacturers (i.e., Philip Morris, R.J. Reynolds, Brown & Williamson and Lorillard) [8], has been instrumental in preventing cigarette smoking in the last several decades [9–11]. For example, the MSA set new advertising, marketing, promotional, and flavoring restrictions to help prevent cigarette smoking uptake among young people [8, 12]. Importantly, the lawsuit also helped to change population perceptions about the cigarette industry, by unveiling industry documents and funding effective cigarette smoking prevention campaigns [8] that disclosed the cigarette industry’s deceptive marketing practices and denormalized the cigarette industry [13–18]. For example, a previous study found that youth and young adults with more exposure to news coverage about litigation of “light” cigarettes were less likely to believe the health “benefits” of “light” cigarettes compared to those with less litigation news exposure [19]. This previous study also found that having inaccurate beliefs about “light” cigarettes was associated with lower perceived risks of cigarette smoking and reduced intention to quit smoking [19]. Overall, this previous study showed that comprehensive awareness of tobacco litigation may impact tobacco-related knowledge and perceptions, and potentially influence tobacco-related behaviors among youth and young adults.

Since the early 2010’s, the cigarette industry has begun acquiring e-cigarette companies (i.e., e-cigarette industry) and making their own e-cigarettes [3]. For example, Vuse [20], the leading e-cigarette brand accounting for a little more than 30% of the U.S. e-cigarette market share in 2022 [21, 22] is manufactured by RJ Reynolds, the maker of Newport and Camel cigarettes [23]. Juul, another leading e-cigarette brand accounting for approximately an additional 30% of the U.S. e-cigarette market share in 2022 [21, 22], was acquired with a 35% stake by Altria [24], the maker of Malboro cigarettes [25], in 2018. Most recently in 2023, Altria announced definitive agreement to acquire full global ownership of NJOY e-cigarettes, including NJOY ACE, the only pod-based e-cigarette with market authorizations from the U.S. Food and Drug Administration as of March 2023 [26]. Thus, this connection between the cigarette and e-cigarette industries makes it unsurprising that the e-cigarette industry has engaged in some of the same predatory activities (e.g., cartoon-like imagery in advertising [27], flavorings) that the cigarette industry was once allowed to use pre-MSA to sell cigarettes to young people [28]. Recent research has shown that being unaware of the connection between the cigarette and e-cigarette industries is associated with more positive perceptions of the e-cigarette industry among young adults, which may potentially lead to e-cigarette use [29]. Additionally, misperceiving that the e-cigarette and cigarette industries were different entities is associated with current e-cigarette use among young adults [29].

While awareness of the MSA and the deceptive marketing practices of the cigarette industry may be well-known to older generations of young adults through effective public education campaigns [9], it is unclear whether the current generation of young adults (i.e., a part of the millennial and generation z) post-MSA era are aware of the MSA and the cigarette industry’s deceptive marketing practices. Given the effectiveness of anti-cigarette industry messaging with preventing cigarette smoking in previous young adult generations [13–18], perhaps educating millennial and generation Z young adults about the deceitful tactics of the cigarette industry and the substantial cigarette-and-e-cigarette industry connection may influence their perceptions about the e-cigarette industry and e-cigarette health risk perceptions to ultimately help prevent e-cigarette use. Although previous research has examined perceptions about industry connections [29], there is a lack of research examining MSA litigation awareness and awareness of cigarette industry practices that can inform e-cigarette prevention research. To help fill these research gaps, we examined associations between awareness of the MSA and cigarette industry practices with e-cigarette industry perceptions and perceived health risks of e-cigarette use among tobacco naïve young adults susceptible to e-cigarette use. We also examined demographic correlates of MSA awareness, awareness of cigarette industry practices, e-cigarette industry perceptions, and perceived health risks of e-cigarette use. Demographic correlates were examined given the targeted marketing of cigarettes to minoritized populations [30], particularly Black individuals [31], and the varying exposure to e-cigarette advertising [32, 33], susceptibility [34], and use behaviors among young adults by demographic characteristics [35, 36]. For our primary analysis, we hypothesized that greater awareness of the MSA and cigarette industry practices may be associated with young adults’ less positive e-cigarette industry perceptions and greater e-cigarette health risk perceptions, which then may be helpful strategies in preventing e-cigarette initiation.

## Methods

Data were from a U.S. sample of tobacco naïve young adults ages 18-30 years-old susceptible to e-cigarette use (*n* = 1,329) recruited and cross-sectionally surveyed through Qualtrics online panel services in August 2021-January 2022. Qualtrics’ online panel participants were recruited from various sources, including website intercept recruitment, member referrals, targeted email lists, gaming sites, customer loyalty web portals, permission-based networks, and social media. Potential participants were invited to participate in the study through Qualtrics panel emails. Qualtrics implemented sampling quotas for race and ethnicity, gender, and educational attainment to improve statistical power for examining associations across these groups. Participants were considered tobacco naïve if they reported never using the following tobacco products, not even one or two puffs/times: e-cigarettes, cigarettes, cigarillos, little filtered cigars, large premium cigars, hookah tobacco, smokeless tobacco and heated tobacco products. Participants were given a description of e-cigarettes similarly to a national tobacco survey [37] and were asked four e-cigarette use susceptibility items (i.e., “Do you think that you will use a vape soon?”; “Do you think that you will use a vape in the next year?”; “Do you think that in the future you might experiment with vapes?”; “If one of your best friends were to offer you a vape, would you use it?”) [38]. Participants were considered susceptible to e-cigarette use if they reported a response (“definitely yes”; “probably yes”; “probably not”; “definitely not”) other than “definitely not” to all four susceptibility items. Of those screened (*n* = 17,831), 1,329 participants were eligible and completed an online survey after providing their informed consent. Participants were compensated according to Qualtrics’ panel provider compensation systems including rewards, membership points, and gift cards. This study received an exempt determination from Institutional Review Board (IRB) review by the National Institutes of Health, Office of IRB Operations.

### Measures

#### Demographic characteristics

Participants reported their demographic characteristics including age (collected and coded as a continuous variable), race and ethnicity (coded as Hispanic [any race]; Black/African American; White; or another race [i.e., Indigenous, Asian, multi-racial, Native Hawaiian or Pacific Islander, or “other” race), gender (coded as man; woman), educational attainment (coded as ≤ high school or GED degree; vocational school or some college; or ≥ a college degree), annual household income (coded as < $35,000; ≥ $35,000 to < $100,000, and ≥ $100,000), and sexual orientation (coded as heterosexual or LGBQ + orientation [lesbian or gay, bisexual, or “something else”]). 

#### Awareness of the Master Settlement Agreement (MSA)

In a single item, participants were asked, “Before today, have you heard of the 1998 Master Settlement Agreement between the U.S. states and cigarette companies?” and reported the extent of their awareness about the MSA. Response and analytic variable categories included: “yes, I have heard of it and know a lot about it”; “yes, I have heard of it, but don’t know much about it”; and “no, I have not heard of it.”

#### Awareness of cigarette industry practices

Participants were asked to report whether they thought cigarette companies engaged in 11 cigarette industry practices (e.g., “Denied under oath that smoking causes lung cancer, even though they had internal scientific evidence”; “Sponsored activities connected to cultural traditions e.g., Native American powwows, Chinese New Year, Cinco de Mayo, African/Black History Month”; “Increased the level of nicotine in cigarettes to keep smokers addicted”; “Added menthol to make cigarettes seem less harsh and more appealing to new smokers and young people”; see supplemental materials for all practices) [39–41]. Response options to each item were “yes”, “no”, or “don’t know”. We recoded responses to represent awareness of each cigarette industry practice (i.e., yes = 1; no/don’t know = 0) and counted the number of practices participants were aware of to create an overall score ranging from 0–11 practices.

#### Electronic cigarette industry perceptions

In eight adapted items [42, 43], participants were asked to report their level of agreement (1 = strongly disagree to 4 = strongly agree) to statements about the e-cigarette industry (i.e., “Electronic vaping companies are responsible for young people vaping”; “Electronic vaping companies are trying to convince the public that vapes are safe”; “Electronic vaping companies are in the business of keeping people hooked on nicotine”; “Electronic vaping companies heavily advertise their products to young people”; see supplemental materials for all e-cigarette industry perception items) (Cronbach’s alpha = 0.88). We reverse coded responses and averaged them to create an overall e-cigarette industry perception score. Higher scores represent more positive perceptions of the e-cigarette industry.

#### Electronic cigarette health risk perceptions

Participants were asked to report the level of likelihood they believed someone would experience 18 potential health harm and addictive risks of e-cigarette use (e.g., “Get exposed to harmful chemicals”; “Harm their brain development”; “Have difficulty quitting [electronic cigarettes]”; see supplemental materials for all e-cigarette health risk perception items). (Cronbach’s alpha = 0.95). Responses ranged from very unlikely = 1 to very likely = 4. We averaged responses to create an overall health risk perception score with higher scores representing higher perceived likelihood of absolute health harm and addictive risks of e-cigarette use.

### Statistical analysis

We used descriptive statistics (e.g., frequencies, proportions, means, and standard deviations) to summarize sample characteristics. We used a multivariable multinomial logistic regression model to examine demographic associations with awareness of the MSA. We also used multivariable linear regression models to examine demographic associations with awareness of cigarette industry practices, e-cigarette industry perceptions, and e-cigarette health risk perceptions. We then assessed multicollinearity between awareness of the MSA and awareness of cigarette industry practices using a multivariable linear regression model, adjusted for demographic characteristics, to examine Variance Inflation Factors (VIF): awareness of the MSA was entered as an independent variable (“Heard, know a lot” category VIF score = 1.7; “heard, don’t know much” category VIF score = 1.4) and awareness of cigarette industry practices was entered as the outcome variable. The VIF scores from this model indicated no significant collinearity [44]. We used a multivariable linear regression model to examine the associations of awareness of the MSA and cigarette industry practices with e-cigarette industry perceptions, adjusting for demographic characteristics. Additionally, we used multivariable linear regression models to assess the associations between awareness of the MSA and cigarette industry practices and e-cigarette industry perceptions with e-cigarette health risk perceptions in two models. In the first model, only awareness of the MSA and cigarette industry practices, as well as demographic characteristics were included. In the second model, e-cigarette industry perceptions was additionally included. This sequential approach was used to avoid potential underestimation of the associations of awareness of the MSA and cigarette industry practices with e-cigarette health risk perceptions [45]. Drawing from the cigarette smoking literature, we chose to include e-cigarette industry perceptions as an independent variable in this model since cigarette industry perceptions is associated with increased smoking health risk perceptions [12]. Overall, there was < 5% missing data for variables of interest, and listwise deletion was used for variables included in the models. All analyses were conducted in SPSS version 28 (Armonk, NY: IBM Corp.).

## Results

Characteristics of the sample are presented in Table 1. Overall, 36.2% had heard of the MSA and knew a lot about it, 24.1% had heard of the MSA, but did not know much about it, and 39.3% did not hear of the MSA. On average, participants were aware of 5.2 (SD = 3.0) of the 11 cigarette industry practices included. Participants also had somewhat negative perceptions of the e-cigarette industry overall, scoring below the mid-point of the scale (Mean = 2.0, SD = 0.63), and perceived health risks from e-cigarette use as likely to happen (Mean = 2.9, SD = 0.69). Awareness of the MSA, awareness of cigarette industry practices, e-cigarette industry perceptions and e-cigarette health risk perceptions by demographic characteristics are also presented in Table 1.

**Table 1:** Sample characteristics by awareness of the Master Settlement Agreement and cigarette industry practices, electronic cigarette industry perceptions, and electronic cigarette health risk perceptions (*N* = 1,329)

**Demographic characteristics**	**Sample**		**Awareness of the Master Settlement Agreement **			**Awareness of cigarette industry practices**	**Electronic cigarette industry perceptions**	**Electronic cigarette health risk perceptions**
			**Heard, know**	**Heard, don’t know**	**Not heard**			
	**% (n)**	**Mean (SD)**	**% (n)**	**% (n)**	**% (n)**	**Mean (SD)**	**Mean (SD)**	**Mean (SD)**
**Overall**	–	–	36.2 (481)	24.1 (320)	39.3 (522)	5.2 (3.0)	2.0 (0.63)	2.9 (0.69)
**Age**	–	24.44 (3.4)	–	–	–	**–**	–	–
**Race and ethnicity**								
Hispanic	14.1 (187)		14.4 (27)	18.7 (35)	66.3 (124)	4.6 (3.4)	2.1 (0.68)	2.8 (0.79)
Black	16.3 (216)		16.7 (36)	21.8 (47)	61.1 (132)	4.0 (3.5)	2.2 (0.70)	2.7 (0.71)
White	61.0 (811)		47.8 (388)	26.3 (213)	25.9 (210)	5.7 (2.7)	1.9 (0.58)	3.0 (0.67)
Other race	8.4 (112)		25.9 (29)	22.3 (25)	49.1 (55)	5.2 (3.2)	2.1 (0.60)	2.9 (0.60)
**Gender**								
Men	48.5 (645)		47.0 (303)	27.3 (176)	25.6 (165)	5.5 (2.8)	2.0 (0.64)	2.9 (0.71)
Women	49.0 (651)		172 (26.4)	21.0 (137)	51.8 (337)	4.9 (3.2)	2.0 (0.62)	2.9 (0.69)
**Educational attainment**								
≤ high school/GED	39.4 (523)		34.6 (181)	21.4 (112)	42.8 (224)	4.5 (3.1)	2.1 (0.66)	2.8 (0.73)
Some college	26.9 (357)		34.5 (123)	26.6 (95)	38.9 (139)	5.4 (3.0)	1.9 (0.54)	3.0 (0.59)
≥ college degree	33.8 (449)		39.4 (177)	25.2 (113)	35.4 (159)	5.9 (2.8)	1.9 (0.65)	2.9 (0.72)
**Annual household income**								
< $35,000	30.1 (400)		7.8 (31)	22.8 (91)	68.0 (272)	4.2 (3.6)	2.3 (0.70)	2.7 (0.73)
≥ $35,000 to < $100,000	39.0 (518)		40.2 (208)	27.4 (142)	32.4 (168)	5.6 (2.9)	1.9 (0.63)	2.9 (0.72)
≥ $100,000	30.9 (411)		58.9 (242)	21.2 (87)	20.0 (82)	5.8 (2.9)	1.8 (0.43)	3.2 (0.52)
**Sexual orientation**								
Heterosexual	86.0 (1143)		40.2 (460)	24.7 (282)	34.6 (396)	5.2 (2.9)	2.0 (0.62)	2.9 (0.70)
LGBQ +	11.4 (152)		13.2 (20)	17.1 (26)	69.1 (105)	5.7 (3.5)	2.0 (0.60)	2.9 (0.59)

Other race category includes individuals identifying as American Indian or Alaska Native, Asian, Native Hawaiian or Pacific Islander, multi-racial or “other”; LGBQ + category includes individuals identifying as lesbian, gay, bisexual or “something else”; SD indicates standard deviation; some n totals for categories within variables do not sum to total sample size due to sporadic missing data (< 5% of cases for any individual variable)

Demographic correlates of awareness of the MSA, awareness of cigarette industry practices, e-cigarette industry perceptions, and e-cigarette health risk perceptions from regression models are presented in Table 2. Results from the multivariable multinomial logistic regression model showed that older participants (vs. younger), men (vs. women), and those with ≤ high school and some college education (vs. college degree) were more likely to report either having heard of the MSA and knowing a lot about it or having heard of the MSA and not knowing much about it relative to not hearing about the MSA. Participants self-identifying as Hispanic, Black, and other race (vs. White participants), with < U.S. $35,000 and ≥ U.S. $35,000 to < U.S. $100,000 annual household income (vs. ≥ U.S. $100,000), and LGBQ + sexual orientation (vs. heterosexual) were less likely to report either having heard of the MSA and knowing a lot about it or having heard of the MSA and not knowing much about it relative to not hearing about the MSA.

**Table 2:** Demographic correlates of awareness of the Master Settlement Agreement and cigarette industry practices, electronic cigarette industry perceptions, and electronic cigarette health risk perceptions (*N* = 1,329)

**Demographic variables**	**Awareness of the Master Settlement Agreement **				**Awareness of cigarette industry practices**		**Electronic cigarette industry perceptions**		**Electronic cigarette health risk perceptions**	
	**Heard, know**^**a**^		**Heard, don’t know**^**a**^							
	**AOR**	**95% CI**	**AOR**	**95% CI**	**B**	**95% CI**	**B**	**95% CI**	**B**	**95% CI**
**Age**	**1.11**	**1.05,1.17**	**1.02**	**0.97,1.07**	-0.01	-0.06,0.05	-0.01	-0.02,0.01	-0.00	-0.02,0.01
**Race and ethnicity**										
Hispanic	**0.29**	**0.17,0.51**	**0.41**	**0.25,0.66**	**-0.55**	**-1.08,-0.18**	0.09	-0.02,0.20	0.03	-0.02,0.15
Black	**0.27**	**0.16,0.46**	**0.45**	**0.29,0.70**	**-1.11**	**-1.61,-0.61**	**0.17**	**0.06,0.27**	-0.05	-0.17,0.07
White	REF	**–**	REF	–	REF	–	REF	–	REF	–
Other race	**0.49**	**0.27,0.89**	**0.63**	**0.36,1.11**	-0.28	-0.90,0.32	0.06	-0.06,0.19	0.06	-0.09,0.20
**Gender**										
Men	**2.95**	**2.12,4.10**	**2.22**	**1.62,3.05**	0.00	-0.34,0.34	0.07	-0.00, 0.14	-0.02	-0.10,0.06
Women	REF	–	REF	–	REF	–	REF	–	REF	–
**Educational attainment**										
≤ high school/GED	**3.64**	**2.40,5.54**	**1.64**	**1.09,2.49**	**-0.99**	**-0.86,-0.01**	0.02	-0.07,0.10	0.02	-0.08,0.12
Some college	**1.63**	**1.08,2.45**	**1.57**	**1.05,2.35**	-0.44	-0.34,0.34	-0.06	-0.14,0.03	**0.14**	**0.04,0.24**
≥ college degree	REF	–	REF	–	REF	–	REF	–	REF	–
**Annual household income**										
< $35,000	**0.06**	**0.04,0.11**	**0.49**	**0.31,0.78**	-0.87	-1.36,0.39	**0.42**	**0.32,0.52**	**-0.48**	**-0.60,-0.37**
≥ $35,000 to < $100,000	**0.47**	**0.33,0.68**	0.85	0.57,1.27	-0.02	-0.40,0.37	**0.18**	**0.10,0.26**	**-0.31**	**-0.40,-0.22**
≥ $100,000	REF	–	REF	–	REF	–	REF	–	REF	–
**Sexual orientation**										
Heterosexual	REF	–	REF	–	REF	–	REF	–	REF	–
LGBQ +	**0.28**	**0.15,0.54**	**0.45**	**0.27,0.77**	**0.91**	**0.35,1.47**	**-0.15**	**-0.26,-0.03**	0.04	-0.10,0.17

^a^indicates that the reference group is “No, I have not heard of it.”; AOR indicates adjusted odds ratios; B indicates unstandardized beta coefficient; 95% CI indicates 95% confidence interval; bolded values indicate statistical significance *p* < 0.05

The multivariable linear regression model with awareness of cigarette industry practices showed that Hispanic participants (vs. White participants), Black participants (vs. White participants) and those with ≤ high school education (vs. college degree) were aware of fewer cigarette industry practices. Participants identifying with LGBQ + sexual orientation (vs. heterosexual) were aware of more cigarette industry practices.

Results from the multivariable linear regression model with e-cigarette industry perceptions revealed that Black participants (vs. White participants) and those with < U.S. $35,000 and ≥  U.S. $35,000 to < U.S. $100,000 annual household income (vs. ≥ U.S. $100,000) had more positive perceptions of the e-cigarette industry, while those identifying with LGBQ + sexual orientation had less positive perceptions of the e-cigarette industry than those identifying with heterosexual sexual orientation.

Lastly, results from the multivariable linear regression model with e-cigarette health risk perceptions showed that participants with some college education perceived greater health risks from e-cigarette use (vs. college degree), while those with those with < U.S. $35,000 and ≥ U.S. $35,000 to < U.S. $100,000 annual household income (vs. ≥ U.S. $100,000) had lower health risk perceptions of e-cigarette use.

Table 3 presents results from the multivariable linear regression models examining associations with the two outcome variables of e-cigarette industry perceptions and e-cigarette health risk perceptions. The multivariable linear regression model that examined associations between awareness of the MSA and cigarette industry practices with e-cigarette industry perceptions, adjusted for demographic characteristics, showed that those who heard of the MSA and knew a lot about it (vs. those who did not hear about the MSA) (B = -0.10 95% CI = -0.19,-0.02) and those who were aware of more cigarette industry practices (B = -0.06, 95% CI = -0.07,-0.05) had less positive perceptions of the e-cigarette industry, whereas those who heard of the MSA, but didn’t know much about it (vs. those who did not hear about the MSA) (B = 0.09, 95% CI = 0.01,0.18) had more positive perceptions of the e-cigarette industry. We also conducted further analysis to compare those who did not hear about the MSA to those who heard of the MSA, but did not know much about it, and results indicated that those who did not hear about the MSA had less positive perceptions of the e-cigarette industry (vs. those who heard of the MSA, but did not know much about it) (B = -0.09, 95% CI = -0.18,-0.01).

**Table 3:** Associations with electronic cigarette industry perceptions and electronic cigarette health risk perceptions (*N* = 1,329)

	**Outcome variables**			
**Independent variables**	**Electronic cigarette industry perceptions**		**Electronic cigarette health risk perceptions**	
	**B**	**95% CI**	**B**	**95% CI**
**Awareness of the Master Settlement Agreement**				
Heard, know a lot	**-0.10**	**-0.19,-0.02**	**0.20**	**0.10,0.30**
Heard, don’t know	**0.09**	**0.01,0.18**	**-0.12**	**-0.22,-0.02**
Not heard of	REF	**–**	REF	**–**
**Awareness of cigarette industry practices**	**-0.06**	**-0.07,-0.05**	**0.04**	**0.03,0.06**
**Electronic cigarette industry perceptions**	–	–	**-0.54**	**-0.60,-0.48**

Multivariable linear regression models adjusted for age, race and ethnicity, gender, educational attainment, annual household income, and sexual orientation; B indicates unstandardized beta coefficient; 95% CI indicates 95% confidence interval; bolded values indicate statistical significance *p* < 0.05. Estimates for awareness of the Master Settlement Agreement and awareness of cigarette industry practices were not adjusted for electronic cigarette industry perceptions, while estimate for electronic cigarette industry perceptions was adjusted for awareness of the Master Settlement Agreement and awareness of cigarette industry practices

The multivariable linear regression models that examined associations between awareness of the MSA, awareness of cigarette industry practices, and e-cigarette industry perceptions with e-cigarette health risk perceptions, adjusted for demographic characteristics, revealed that those who had heard of the MSA and knew a lot about it (vs. those who did not hear about the MSA) (B = 0.20, 95% CI = 0.10,0.30) and those who were aware of more cigarette industry practices (B = 0.04, 95% CI = 0.03,0.06) had higher perceived health risks of e-cigarette use, while those who had heard of the MSA, but didn’t know much about it (vs. those who did not hear about the MSA) (B = -0.12, 95% CI = -0.22,-0.02) had lower perceived health risks of e-cigarette use. Additionally, we conducted further analysis to compare those who did not hear about the MSA to those who heard of the MSA, but did not know much about it and found that those who did not hear about the MSA had higher e-cigarette risk perceptions (B = 0.12, 95% CI = 0.02,0.22). Following, when we entered e-cigarette industry perceptions into the original model with those who did not hear about the MSA as the reference group for the awareness of the MSA variable, we found that more positive perceptions of the e-cigarette industry (B = -0.54, 95% CI = -0.60,-0.48) was also associated with lower perceived health risks of e-cigarette use.

## Discussion

Despite previous young adult generations’ in the late 1990’s and early 2000’s knowing about the MSA and cigarette industry practices through cigarette smoking prevention efforts like the Truth Campaign [17], it was unknown how much this current young adult generation in the early 2020’s are aware of the MSA and previous cigarette industry practices. We hypothesized that greater awareness of the MSA and more cigarette industry practices could in turn be associated with less positive perceptions of the e-cigarette industry and higher perceived health risks of e-cigarette use. This is an important consideration given that low e-cigarette health risk perceptions is associated with initiating e-cigarette use [46]. We found that while hearing about the MSA and knowing a lot about it was associated with less positive perceptions of the e-cigarette industry and higher perceived risks of e-cigarette use, hearing about the MSA *but not knowing much about it *was associated with more positive perceptions of the e-cigarette industry and lower perceived risks of e-cigarette use, relative to not hearing about the MSA at all. Though these results about hearing about the MSA but not knowing much about it were surprising, these results align with previous research that suggest having more exposure to litigation may be informative and help shape accurate beliefs [19]. Perhaps, simply being aware of the term “Master Settlement Agreement” without knowing about the details of the litigation may leave young adults with ambiguity about the MSA, which may potentially lead to dangerous misperceptions and misinterpretations of the “MSA”, and in turn influence their e-cigarette-related perceptions. This speculation is supported by our finding that greater awareness of more cigarette industry practices is associated with less positive perceptions about the e-cigarette industry and higher perceived health risks of e-cigarette use. Perhaps, the importance of being aware of the MSA and cigarette industry practices is grounded in cumulative understanding of the cigarette industry’s history and practices, and how much young adults know about these aspects of the cigarette industry.

Overall, the potential impact of anti-e-cigarette industry messaging to prevent e-cigarette use has been mixed. While a previous study found that messaging themed as industry targeting performed less effectively than other messaging themes examined [47], there are some previous research suggesting the potential utility of anti-e-cigarette industry messaging to prevent e-cigarette use. For example, exposure to anti-e-cigarette industry health communication was associated with more anti-industry attitudes among young adults in a previous study [48]. Another previous study also found that anti-e-cigarette industry messaging connecting the e-cigarette industry to “Big Tobacco” was associated with increased perceived health risks of e-cigarette use, less intentions to use e-cigarettes, and more support for e-cigarette policy control [49]. Findings from the current study build on previous research by providing insights on potentially helpful message content to improve the potential effectiveness of anti-industry e-cigarette prevention messaging. Future research should examine whether messaging content about the longstanding history of the cigarette industry’s deceptive practices and the MSA can impact perceptions about the e-cigarette industry, e-cigarette health risk perceptions, and ultimately susceptibility and initiation of e-cigarettes among young adults. Future research is also needed to understand how awareness of cigarette industry practices may influence use of other tobacco products among young adults.

Increasing awareness about the MSA and cigarette industry practices may be promising given that sampled tobacco naïve young adults susceptible to e-cigarette use had little awareness about the MSA and the deceptive practices of the cigarette industry. Specifically, 36% of the sample reported hearing about the MSA and knowing a lot about it. On average, tobacco naïve young adults susceptible to e-cigarette use in this study were also aware of five of the 11 cigarette industry practices we included. These findings suggest the potential for increasing knowledge to help promote attitudinal changes and prevent dangerous misperceptions about the cigarette and e-cigarette industries. Overall, efforts to inform the current young adult generation about the cigarette industry’s deceptive practices and the revival of the cigarette industry’s playbook with e-cigarettes may help change perceptions that support susceptibility.

Findings from this sample also suggest that racial and ethnic young adults, young adults with ≤ high school/GED and some college education, young adults with < U.S. $35,000 annual household income, and young adults identifying with LGBQ + sexual orientation may most benefit from efforts to increase awareness about the MSA. Additionally, increasing awareness about cigarette industry marketing practices may be especially important for Hispanic and Black young adults and those with lower education (i.e., ≤ high school/GED vs. ≥ college degree) who had less awareness of these practices in this sample. Such efforts can have important tobacco control implications, especially given that these populations have been and continue to be profiled by the cigarette industry [50–52].

This study has a few limitations. Although this study includes a U.S. sample of tobacco naïve young adults susceptible to e-cigarette use with quota sampling to improve statistical power to examine demographic correlates, the sample was recruited through online panel non-probability sampling approaches and may not be representative of U.S. young adults susceptible to e-cigarette use and may not be generalizable to other young adults such as those who use tobacco products. We were also unable to include gender minority participants in the analysis due to small sample sizes. It is important for future studies to examine awareness of the MSA and cigarette industry practices and their associations with e-cigarette-related perceptions among gender minority populations. Additionally, we did not assess what participants knew about the MSA and their knowledge about the connection between the two industries. Since awareness of the MSA and cigarette industry practices may be subject to social desirability bias, awareness in this study may be overestimated (awareness of the MSA = 36.2%; awareness of cigarette industry practices = 5.2 out of 11 included). Nonetheless, this study informs future e-cigarette prevention research with a potential messaging content strategy. Future research is needed to better understand how raising awareness about the MSA and cigarette industry practices can potentially change perceptions about the e-cigarette industry and e-cigarette health risk perceptions, and potentially help prevent initiation and use.

## Conclusions

This study examined awareness of the 1998 Master Settlement Agreement and cigarette industry practices among millennial and generation Z young adults aged 18- to 30 years-old who were either very young or not yet born during the height of cigarette smoking prevention efforts exposing the cigarette industry’s practices. We found that young adults susceptible to e-cigarette use who had heard of the MSA and knew a lot about it and those who were aware of more cigarette industry practices had less positive perceptions of the e-cigarette industry and higher perceived risks of e-cigarette use. Importantly, our findings also suggest that hearing of the MSA, but not knowing much about it may have negative implications for young adults’ e-cigarette-related perceptions. Thus, future research should examine whether informing young adults’ about the cigarette industry’s longstanding history of deceptive marketing practices pre-MSA, and the cigarette industry’s connection to the e-cigarette industry, may be a potentially impactful e-cigarette prevention messaging strategy.

## Supplementary Information

**Additional file 1.**

## Data Availability

The datasets generated and/or analysed during the current study are not publicly available due to the proprietary nature of the data but are available from the corresponding author on reasonable request.
