# Supplementary material for: U.S. young adults’ awareness of the Master Settlement Agreement and cigarette industry practices and their associations with electronic cigarette industry and health risk perceptions
Source: BMC Public Health. 2023 Mar 31;23:615. doi: 10.1186/s12889-023-15520-2 (PMC10064685; doi:10.1186/s12889-023-15520-2)
Supplement: Supplementary file 1 — Additional file 1. [file 12889_2023_15520_MOESM1_ESM.docx]

**Supplemental Materials**

**1. Awareness of Cigarette Industry Practices**

| **Do you think cigarette companies have engaged in the following activities?** | **Yes** | **No** | **Don’t Know** |
| --- | --- | --- | --- |
| Denied under oath that smoking causes lung cancer, even though they had internal scientific evidence |  |  |  |
| Compared smoking to habitual shopping or using the internet, while knowing nicotine was as addictive as cocaine and heroin |  |  |  |
| Had a target age group of men 12-17 and women 12-34  Sponsored activities connect to cultural traditions (e.g., Native American powwows, Chinese New Year, Cinco de Mayo, African/Black History Month) |  |  |  |
| Looked at ways to keep youth smoking and not quit |  |  |  |
| Used sporting event sponsorships to market to youth |  |  |  |
| Increased the level of nicotine in cigarettes to keep smokers addicted |  |  |  |
| Marketed “lighter” and “low tar” cigarettes as safer than regular cigarettes to mislead smokers, even though they knew that these alternative cigarettes were even more harmful than regular cigarettes |  |  |  |
| Set up independent foundations designed to confuse the public about the dangers of secondhand smoke |  |  |  |
| Added menthol to make cigarettes seem less harsh and more appealing to new smokers and young people |  |  |  |
| Hid unfavorable research results to mislead the public |  |  |  |

**2. Electronic Cigarette Industry Perceptions**

| **Based on what you believe, please rate your level of agreement for each statement.** | **Strongly disagree** | **Disagree** | **Agree** | **Strongly agree** |
| --- | --- | --- | --- | --- |
| Electronic vaping companies are responsible for young people vaping. |  |  |  |  |
| Electronic vaping companies are trying to convince the public that vapes are safe. |  |  |  |  |
| Electronic vaping companies are in the business of keeping people hooked on nicotine. |  |  |  |  |
| Electronic vaping companies use the same practices (e.g., product development, marketing) as big tobacco companies. |  |  |  |  |
| All the big tobacco companies are in the electronic vaping business. |  |  |  |  |
| Electronic vaping companies try to brand their products as modern devices rather than tobacco products. |  |  |  |  |
| The look and feel of electronic vapes are designed with young people in mind. |  |  |  |  |
| Electronic vaping companies heavily advertise their products to young people. |  |  |  |  |

**3. Electronic Cigarette Health Risk Perceptions**

| **How likely would it be for someone to experience the following if they were to use vapes?** | **Very unlikely** | **Unlikely** | **Likely** | **Very**  **likely** |
| --- | --- | --- | --- | --- |
| Get exposed to harmful chemicals |  |  |  |  |
| Start coughing |  |  |  |  |
| Have trouble catching their breath |  |  |  |  |
| Get chronic obstructive pulmonary disease (i.e., COPD) |  |  |  |  |
| Have heart problems (e.g., heart attack) |  |  |  |  |
| Get oral (mouth) cancer |  |  |  |  |
| Get lung cancer |  |  |  |  |
| Get wrinkles |  |  |  |  |
| Harm their brain development |  |  |  |  |
| Use other tobacco products (e.g., cigarettes, cigars, hookah tobacco, smokeless tobacco) in the future |  |  |  |  |
| Use marijuana in the future |  |  |  |  |
| Use drugs or other substances (e.g., heroin, cocaine, methamphetamines) in the future |  |  |  |  |
| Be controlled by nicotine addiction |  |  |  |  |
| Have difficulty quitting |  |  |  |  |
| Hurt their lungs |  |  |  |  |
| Look awkward |  |  |  |  |
| Become addicted to vaping |  |  |  |  |
| Feel bad taste |  |  |  |  |
